# Survival of *Burkholderia pseudomallei *in Water

**DOI:** 10.1186/1756-0500-1-11

**Published:** 2008-05-07

**Authors:** Richard A Moore, Apichai Tuanyok, Donald E Woods

**Affiliations:** 1 Department of Microbiology and Infectious Diseases, Faculty of Medicine, University of Calgary Health Sciences Centre, Calgary, Alberta, T2N 4N1, Canada; 2 Microbial Genetics and Genomics Center, Northern Arizona University, Flagstaff, Arizona, 86011, USA

## Abstract

**Background:**

The ability of *Burkholderia pseudomallei *to survive in water likely contributes to its environmental persistence in endemic regions. To determine the physiological adaptations which allow *B. pseudomallei *to survive in aqueous environments, we performed microarray analyses of *B. pseudomallei *cultures transferred from Luria broth (LB) to distilled water.

**Findings:**

Increased expression of a gene encoding for a putative membrane protein (BPSL0721) was confirmed using a *lux*-based transcriptional reporter system, and maximal expression was noted at approximately 6 hrs after shifting cells from LB to water. A BPSL0721 deficient mutant of *B. pseudomallei *was able to survive in water for at least 90 days indicating that although involved, BPSL0721 was not essential for survival. BPSL2961, a gene encoding a putative phosphatidylglycerol phosphatase (PGP), was also induced when cells were shifted to water. This gene is likely involved in cell membrane biosynthesis. We were unable to construct a PGP mutant suggesting that the gene is not only involved in survival in water but is essential for cell viability. We also examined mutants of polyhydroxybutyrate synthase (*phb*C), lipopolysaccharide (LPS) oligosaccharide and capsule synthesis, and these mutations did not affect survival in water. LPS mutants lacking outer core were found to lose viability in water by 200 days indicating that an intact LPS core provides an outer membrane architecture which allows prolonged survival in water.

**Conclusion:**

The results from these studies suggest that *B. pseudomallei *survival in water is a complex process that requires an LPS molecule which contains an intact core region.

## Background

*Burkholderia pseudomallei *is the causative agent of melioidosis, a disease endemic to southeast Asia, northern Australia and temperate areas that lie near the equator[1]. The disease is classified by acute, subacute and chronic illnesses and often mistaken for malaria, plague, pneumonia and miliary tuberculosis [2]. The acute form of the disease is a septicemic illness and is often fatal despite antibiotic treatment. Subacute meliodosis often results in multi-organ involvement, systemic abscess formation and bacteremia [3]. Chronic meliodosis often is detected only after post mortem examination or by activation of other forms of the disease as a result of a traumatic event [4]. Infection most likely occurs as a result of aspiration, inhalation or by exposure to contaminated soil or water [5,6].

*B. pseudomallei *is able to survive in soils in endemic areas however recovery is dependent upon moisture content [7]. Previous studies have demonstrated the ability of *B. pseudomallei *to survive in double distilled water for 3 years [8] and follow up studies on the same culture have shown that viable cells still exist after 17 years (Dr. Vanaporn Wuthiekanun, personal communication). Water supplies in Australia have been connected to meliododosis cases [9-11]illustrating the threat that long term survival in water may pose to water supplies.

While *B. pseudomallei *survival in water is well documented, the molecular mechanisms which allow *B. pseudomallei *to survive in water are poorly understood. In the present studies we examined a number of possible factors that may contribute to this survivability.

## Methods

Bacterial strains used in this study are listed in Table 1. Cultures were grown at 37° in Luria-Bertani (LB) broth or on LB agar plates. Antibiotics, when required, were used at the following concentrations: gentamicin (Gm), 25 μg/ml; polymyxin B sulfate (PMB), 100 μg/ml; carbenicillin (CB), 100 μg/ml.

**Table 1:** Bacterial strains and plasmids used in this study.

Strain or plasmid	Description	Reference or source
Strains		
*E. coli *TOP10	F-mcrA Δ(mrr-hsdRMS-mcrBC)Φ80lacZΔM15 ΔlacX74 deoR recA1 araΔ139 Δ(ara-leu)7697	Invitrogen
*S. aureus *ATCC 13709		American Type Culture Collection
*P. aeruginosa *PAO1		American Type Culture Collection
B. pseudomallei		
1026b	Clinical isolate; Km^r ^Gm^r ^Sm^r ^Pm^r ^Tc^s ^Tp^s^	[16]
DD503	1026b derivative; allelic exchange strain; Δ(*amrR-oprA*) (Km^s ^Gm^s ^Sm^s^) *rpsL *(Sm^r^)	[17]
SZ210	DD503 derivative; Δ*wcbB*	[18]
SR5	wcbB::Tn5OT182 Tcr	[14]
MB301	DD503 derivative; *waaE::dhfrIIb*-p15AoriV; Zeo^r^	[19]
SRM117	1026b derivitive; wbiI::Tn5-OT182; Tc^r^	[20]
RM330	DD503 derivative; *phaC*::pGSV3-*lux*	This study
RM0721	DD503 derivative; BPSL0721::pGSV3-*lux*	This study
B. mallei		
ATCC 23344	Type strain genome sequence complete	American Type Culture Collection
Plasmids pGSV3-*lux*	Mobilizable suicide vector containing *lux *operon from *Photorhabdus luminescens*; OriT; Gm^r^	[12]
pCR2.1-TOPO	Topoisomerase-mediated cloning vector; Ap^r ^Km^r^	Invitrogen

Overnight cultures of bacteria were grown in 3 ml of LB in 15 ml plastic tubes. Cells were washed once in sterile distilled, deionized water and an aliquot added to 40 ml of sterile distilled, deionized water to a final concentration of approximately 10^6 ^colony forming units (CFU) per ml. Plate counts were performed immediately after the initial dilution and subsequently at various time points. All plate counts were done in duplicate using LB agar plates without antibiotics.

Mutagenesis of the *B. pseudomallei *poly (3)-hydroxybutyrate synthase gene (*phbC *or BPSL1534) and BPSL0721 was achieved using the *lux*-based suicide vector pGSV3-*lux *as previously described (Moore, *et al*., 2004). Briefly, a 549 bp internal region of the *B. pseudomallei *PHB synthase gene, *phbC *was PCR amplified using the primers GCACAAGACGTGGGACGAC (Forward) and ATGCGCGACAGATCGACGGG (Reverse). For BPSL0721, a 424 bp internal region was amplified using the primers TGTGGATGTCGCAGATGC (Forward) and ATGAAGCGGGTCTTCGACG (Reverse). The amplicons were cloned into pCR2.1-TOPO (Invitrogen) and subsequently excised with *Eco*R1 and cloned into *Eco*R1 digested, CIP treated, pGSV3-*lux*. The resulting plasmids, pGSV-*lux-phbC *and pGSV-*lux*-0721 were electroporated into *E. coli *SM10 λ pir and subsequently conjugated into *B. pseudomallei *DD503. Transconjugants were identified by selection on LB-Gm, PMB agar and tested for *lux *activity by screening for light production using 50–200 ul of an overnight culture in a SystemSure luminometer (Nova Biomedical, Waltham, MA). Integration of pGSV-*lux-phbC *into *phbC *and BPSL0721 was confirmed by PCR using a reverse primer internal to the *luxC *gene on the vector and a forward primer located at the 5' end of the *phbC *and BPSL0721 genes and upstream of the forward primers used to generate the internal fragments originally cloned into pGSV3-*lux*.

Transmission electron microscopy was performed on *B. pseudomallei *DD503 and the *phbC *mutant, RM330. One ml of cells was grown overnight in LB was centrifuged in a microcentrifuge. The supernatant was removed, and the cell pellet was subjected to a series of dehydration steps and finally cast in epoxy resin. Layers of the epoxy resin pellet were mounted on support grids and observed by transmission electron microscopy (University of Calgary Imaging Facility).

Microarray analysis was performed using a previously described low density DNA oligo-microarray [12]. Total bacterial RNA was isolated from two 100 ml cultures grown overnight in LB. Cells were harvested by centrifugation and washed once with sterile water. Both pellets were resuspended in 100 ml of sterile water and one was treated immediately with RNA*later *(Invitrogen), centrifuged and the cell pellet frozen. The remaining flask was allowed to shake at 37° for one hour at which time RNA*later *was added, the cells centrifuged and the cell pellet frozen. Subsequently, cell pellets were thawed and RNA isolated as described previously [12].

## Results

**Figure 1:**
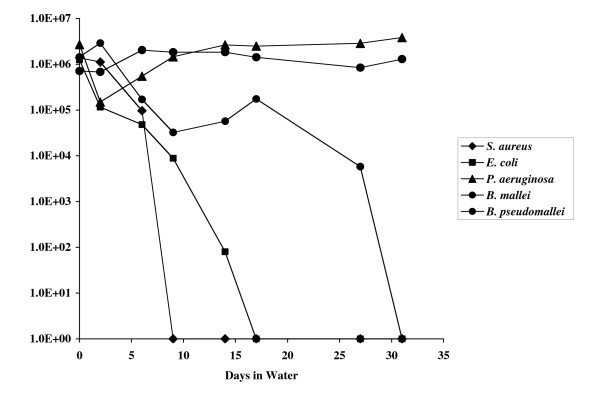
Survival of various bacteria in water. Survival kinetics of bacteria suspended in water. Bacteria were grown overnight in LB. Cells were centrifuged and washed once in water and an aliquot used to make a 40 ml suspension of approximately 10^6 ^CFU/ml in water. Plate counts were done at various time points to determine viable cells remaining.

Different bacteria were compared in terms of their ability to survive in water. These included *B. pseudomallei*, *B. mallei*, *P. aeruginosa*, *E. coli *and *S. aureus*. As seen in Fig. 1, all of the strains, except for *B. pseudomallei *and *P. aeruginosa*, died within 30 days when suspended in water.

**Figure 2:**
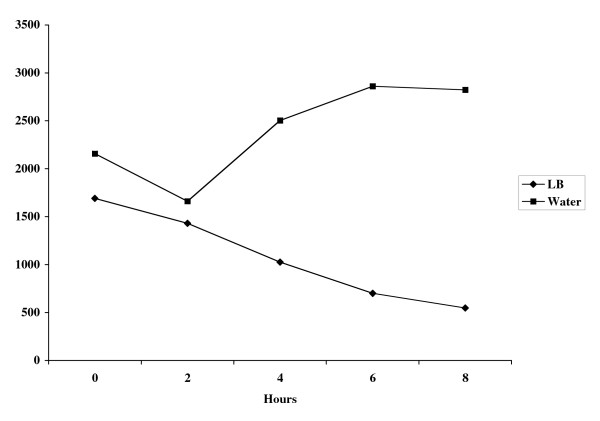
*lux*-mediated light prodution in *B. pseudomallei *RM0721. RM0721 was grown overnight and washed once in water. Cells were resuspended in either LB or water and light production and OD_600 _measured at indicated time points.

**Figure 3:**
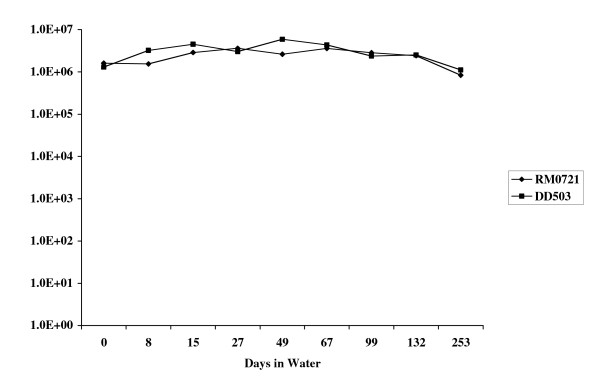
Survival of *B. pseudomallei *RM0721 in water. Mutant RM0721 was grown overnight in LB and cells were washed in water and used to make a 40 ml suspension of approximately 10^6 ^CFU/ml in water. Plate counts were done at various time points to determine viable cells remaining. A similar tube was prepared using DD503 for comparison.

Microarray analysis was performed on *B. pseudomallei *cells that had been transferred from LB broth to sterile water for a period of one hour. The results of this analysis identified a number of genes that were up-regulated as a result of exposure to water (Table 2). These genes include a variety of genes involved in energy metabolism and membrane biosynthesis as well as a number of hypothetical proteins. The suicide vector pGSV3-lux was used to insertionally inactivate one of these genes, BPSL0721, to determine if this gene was essential for survival in water. The resulting mutant, RM0721, was examined further and was shown to produce *lux*-mediated light when shifted from LB to water indicating expression of the BPSL0721 gene under these conditions (Fig. 2). Light production in the mutant peaked at approximately 6 hours after being transferred to water and confirmed microarray data which indicated that this gene was expressed when cells were shifted to water. RM0721 was not, however, impaired in its ability to survive in water (Fig. 3) indicating that the gene was involved but not essential for water survival.

**Table 2:** *Burkholderia pseudomallei *genes up-regulated in water,

BPSL2218	Hypothetical protein
BPSL1944	InfC, translational protein
BPSL2961	putative phosphatidylglycerophosphatase
BPSL1854	hypothetical protein
BPSL1600	putative cytochrome c-related lipoprotein
BPSL0721	putative membrane protein
BPSL0106	putative exported protein
BPSL1723	hypothetical protein
BPSL0313	glmU, bifunctional glmU protein [includes: UDP-N-acetylglucosamine pyrophosphorylase; glucosamine-1-phosphate N-acetyltransferase]
BPSL1614A	putative iron-sulphur protein
BPSL1391	putative phage related protein (partial)
BPSL0660	pdxA, 4-hydroxythreonine-4-phosphate dehydrogenase
BPSL0381	dsbA, thioldisulfide interchange protein
BPSL2772	kdsA, putative 2-dehydro-3-deoxyphosphooctonate aldolase
BPSL0974	conserved hypothetical protein
BPSL1797	putative ABC transport system, membrane protein
BPSL1294	conserved hypothetical protein

BPSL2961, a gene encoding a putative phosphatidylglycerol phosphatase (PGP), was also induced when cells were shifted to water. This gene is likely involved in cell membrane biosynthesis (glycerophospholipid metabolism pathway). We were unable to construct a PGP mutant suggesting that the gene was not only involved in survival in water but is also essential for cell viability.

**Figure 4:**
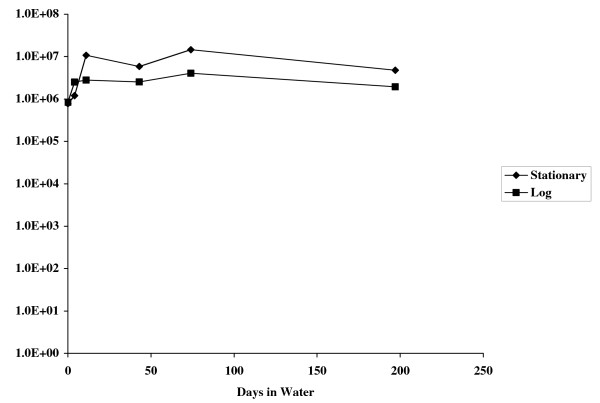
Survival of *B. pseudomallei *in water at different growth phases. *B. pseudomallei *cultures were grown to either mid log phase (OD_600_~0.5) or overnight to stationary phase. Cells were washed in water and used to make a 40 ml suspension of approximately 10^6 ^CFU/ml in water. Plate counts were done at various time points to determine viable cells remaining.

To determine if growth phase effected the ability of *B. pseudomallei *to survive in water, cells taken from log and stationary phase growth were compared in terms of their ability to survive in water. As shown in Fig. 4, there was no apparent difference in survival between cell taken from log or stationary phase growth.

**Figure 5:**
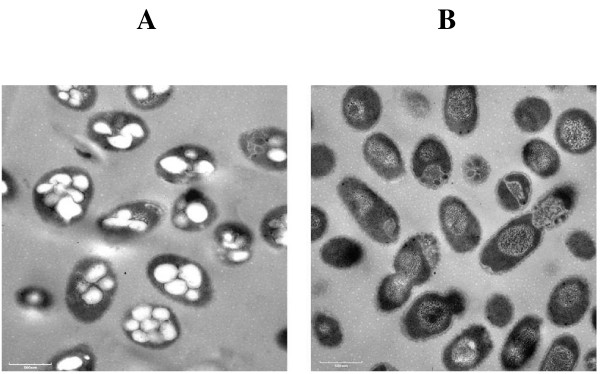
Polyhydroxybutyrate inclusions. Transmission electron micrograph *of B. pseudomallei *showing intracellular inclusions of PHB (white globules) in *B. pseudomallei *DD503 (A) and lack of PHB inclusions in the *phaC *mutant RM330 (B).

Polyhydroxybutyrate (PHB) is a storage compound synthesized by a variety of gram-negative bacteria and has been shown to be involved in water survival in *Legionella pneumophila*[13]. To determine if PHB was required for water survival in *B. pseudomallei *a PHB mutant was constructed. Insertional inactivation of the *phbC *gene using pGSV3-*lux *resulted in a mutant which no longer synthesized PHB as indicated by electron microscopy (Fig. 5). The mutant, RM330, was compared to the parent strain for its ability to survive in water. RM330 remained viable after 300 days in water indicating that PHB is not essential for water survival in *B. pseudomallei *(Additional file 1).

The role of LPS and capsule was examined by testing the ability of various mutants to survive in water. The mutants tested are shown in Table 1 and included a capsule mutant, an oligosaccharide deficient mutant, a double mutant lacking capsule and LPS oligosaccharide and a LPS core deficient mutant. Mutants lacking capsule, LPS oligosaccharide or both were not affected in their ability to survive in water. In contrast, a mutant lacking both oligosaccharide and LPS core died in water by 200 days indicating that intact core is required for long term survival of *B. pseudomallei *in water (Additional file 2).

## Discussion

A water environment presents unique challenges for the bacterial cell. Water lacks the nutrients found in other environments and provides an extreme hypo-osmotic condition. To survive for long periods of time the organism must be able to deal with the metabolic challenges resulting from lack of substrates normally used for maintenance and growth and must be able to adapt to low osmolarity. The fact that this environment requires special adaptations is illustrated by our observation that *E. coli*, *S. aureus *and notably, *B. mallei*, were unable to survive in this environment for prolonged periods.

A variety of factors contribute to the ability of *B. pseudomallei *to survive in the environment. These include a versatile metabolic capacity which allows the use of a variety of carbon sources for growth, the ability to live inside other microorganisms including protoza and fungi and the ability to tolerate a wide range of environmental conditions including a variety of soils and water environments such as those associated with rice farming and seasonal flooding. The ability of *B. pseudomallei *to survive in aqueous environments is a major factor in the survival and persistence of this organism in environments where meliodosis remains endemic.

We tested a number of hypotheses which we thought might contribute to *B. pseudomallei *survival in water. These included an examination of growth phase, PHB, capsule and LPS and the product of one gene that was shown to be up-regulated in water via microarray analysis. We examined cells grown in log and stationary phase hypothesizing that perhaps stationary phase cells had made the necessary modifications to the cell which would allow survival in water whereby cell in log phase had not. We found that cells taken from either growth phases were equally able to survive in water indicating that cell growth phase was not important or that the cells, in either log or stationary phase, must undergo some adaptation upon entering a water environment.

PHB has been shown to be involved in water survival in *Legionella pneumophila *[12] and would seem not unreasonable to play a role in water survival in *B. pseudomallei*. We found no significant loss in viability in a PHB deficient mutant, RM330, in water suggesting that in *B. pseudomallei*, PHB may not be critical as an energy source for long term survival. It is possible however, that PHB has an important role in other situations such as intracellular survival in protozoa and fungi and possibly human cells as well.

We found that *B. pseudomallei *capsule I [14]was not required for survival in water. In contrast, capsule I is essential for *B. pseudomallei *virulence in hamsters where it serves to evade complement killing. That capsule is not solely required for water survival is further illustrated by the fact that *B. mallei*, which doesn't survive in water, expresses the same capsule as *B. pseudomallei*[15].

The mutant MB301, defective in core LPS oligosaccharide, showed a marked loss of viability by 200 days. This mutation also confers sensitivity to polymyxin B, a cationic antibiotic which is normally ineffective against *B. pseudomallei *at levels up to 100 mg/ml suggesting a loss of outer membrane integrity as a result of altered outer membrane architecture. LPS oligosaccharide is not essential for water survival suggesting that, like capsule, the molecule probably does little to help maintain the membrane integrity apparently required for long term water survival.

While this study identifies at least one component required for long term survival in water, it is probable that *B. pseudomallei *undergoes a number of other modifications which allow it to survive for years in nutrient poor, low osmolarity conditions. It is likely that membrane modifications occur as suggested by the up regulation of a putative phosphatidylglycerol phosphatase (BPSL2961). Further, energy metabolism is likely altered as indicated by the up-regulation of a putative cytochrome C related lipoprotein (BPSL1600) and a putative iron-sulfur protein (BPSL1615). In addition, the aqueous environment may trigger a number of cell signals that regulate the various modifications required for long term survival. Understanding how *B. pseudomallei *is able to survive in water may provide a better understanding of the organisms environmental persistence in endemic areas and may provide public health strategies for those living in these areas.

## Authors' contributions

RAM performed all of the mutant construction and water experiments. AT performed the microarray analyses. DEW contributed to experimental design and assisted in the preparation of the manuscript. All authors read and approved the final manuscript.

## Supplementary Material

Additional file 1 Survival of a *B. pseudomallei phaC *mutant in water. *B. pseudomallei *RM330 and DD503 were grown overnight in LB. Cells were centrifuged and washed once in water and an aliquot used to make a 40 ml suspension of approximately 10^6 ^CFU/ml in water. Plate counts were done at various time points to determine viable cells remaining. Click here for file

Additional file 2 Effect of capsule and LPS mutations on water survival. *B. pseudomallei *mutants lacking capsule (SZ210), LPS oligosaccharide (SRM117), capsule and LPS oligosaccharide (SR5) and LPS core (MB301) were tested for their ability to survive in water as described in text. Click here for file
