# Supplementary material for: Survival of Burkholderia pseudomallei in Water
Source: BMC Res Notes. 2008 May 7;1:11. doi: 10.1186/1756-0500-1-11 (PMC2518269; doi:10.1186/1756-0500-1-11)
Supplement: Supplementary file 1 [file 1756-0500-1-11-S1.doc]

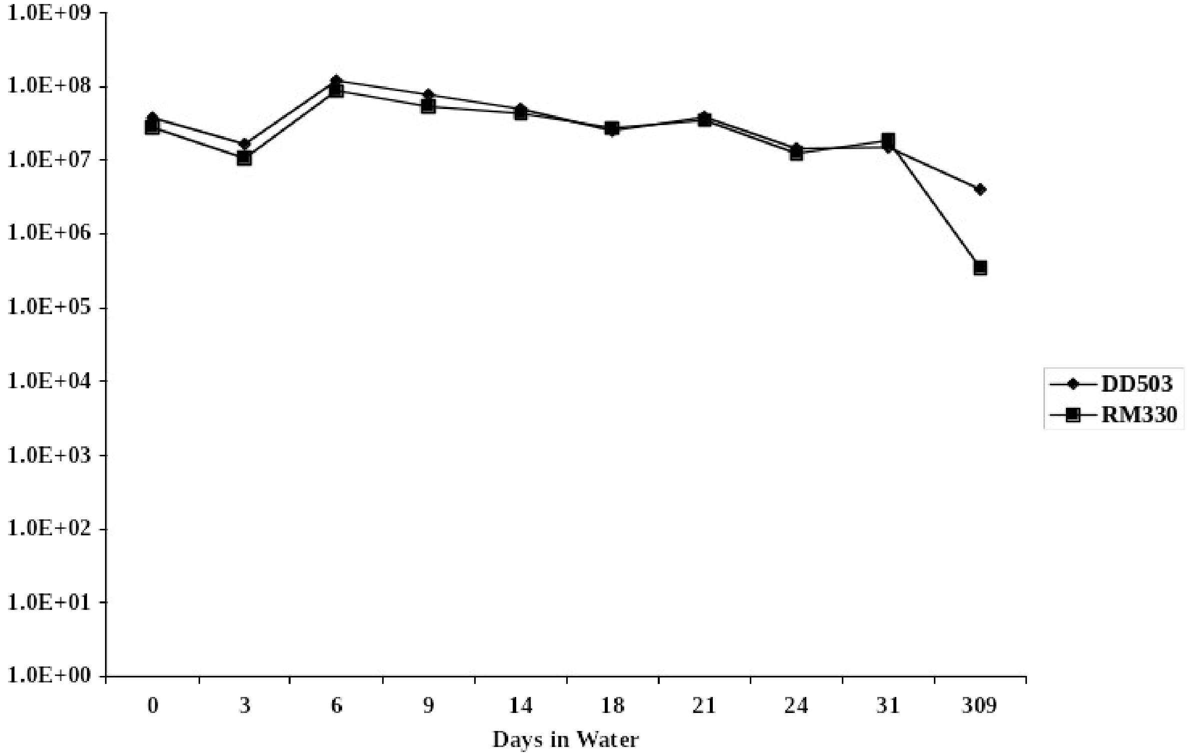

1.0E+09
1.0E+08
1.0E+07
1.0E+06
1.0E+05
1.0E+04
1.0E+03
1.0E+02
1.0E+01
1.0E+00
0
3
6
9
14
18
21
24
31
309
Days in Water
DD503
RM330
