# Supplementary material for: Survival of Burkholderia pseudomallei in Water
Source: BMC Res Notes. 2008 May 7;1:11. doi: 10.1186/1756-0500-1-11 (PMC2518269; doi:10.1186/1756-0500-1-11)
Supplement: Supplementary file 2 [file 1756-0500-1-11-S2.doc]

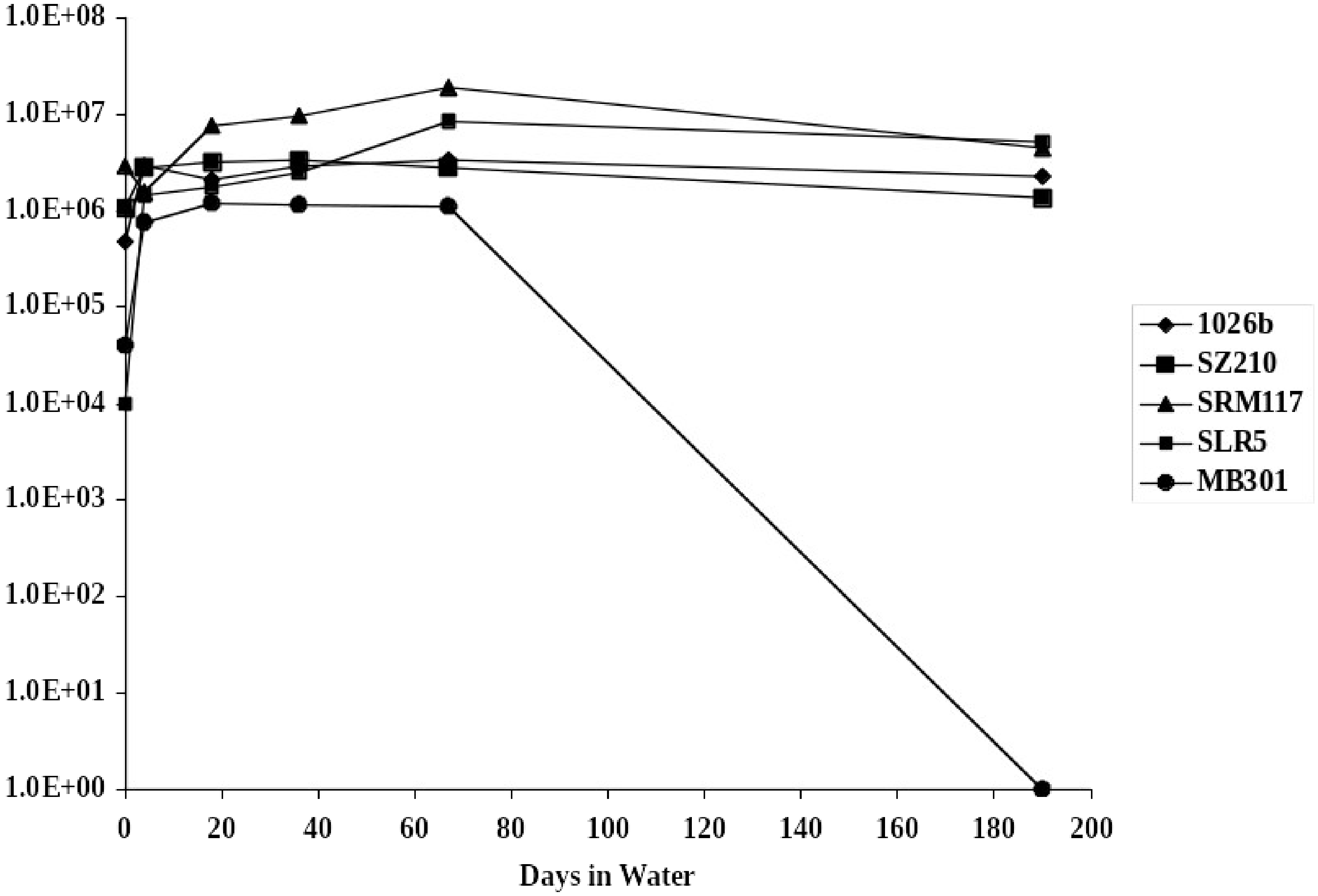
1.0E+08
1.0E+07
1.0E+06
1.0E+05
1.0E+04
1.0E+03
1.0E+02
1.0E+01
1.0E+00
0
20
40
60
80
100
120
140
160
180
200
Days in Water
1026b
SZ210
SRM117
SLR5
MB301
